# Walking Outdoors during Seminars Improved Perceived Seminar Quality and Sense of Well-Being among Participants

**DOI:** 10.3390/ijerph15020303

**Published:** 2018-02-09

**Authors:** Olle Bälter, Björn Hedin, Helena Tobiasson, Susanna Toivanen

**Affiliations:** 1 Department of Media Technology and Interaction Design, KTH—Royal Institute of Technology, SE-100 44 Stockholm, Sweden; bjornh@kth.se (B.H.); tobi@kth.se (H.T.); 2 School of Health, Care, and Social Welfare, Mälardalen University, SE-721 23 Västerås, Sweden; susanna.toivanen@mdh.se

**Keywords:** teaching methods, higher education, seminars, blended learning, outdoor physical activity, well-being

## Abstract

Low levels of physical activity and sedentary behaviour are a growing health problem globally. Physical inactivity is associated with increased risk of numerous ailments, cardiovascular disease and mortality. Our primary aim was to perform a feasibility study on how to incorporate physical activity among students and teachers in regular teaching activities. The second aim was to investigate how students and teachers perceived the differences between outdoor walking seminars and regular indoor seminars. By transforming an on-campus course into a blended course, we were able to conduct seminars outdoors in nearby nature while walking. These walking seminars were evaluated among 131 students and nine teachers leading the walking seminars. The responses to the student survey and teacher interviews indicate that discussions, sense of well-being and the general quality of the seminar improved, regardless of how physically active participants were the rest of the time. The study shows one way to increase physical activity with small means; in our case, a reorganization of how we prepared for the seminars which allowed for walking discussions.

## 1. Introduction

The ancient Greeks walked around outdoors while teaching and learning, both in Plato’s Academia and Aristotle’s Lyceum. Nowadays, however, a large part of the Western population has an indoor, sedentary manner of learning and working. Sedentary lifestyles and low levels of physical activity are two risk factors, increasingly highlighted in medical studies as a major public health problem, with increased risk for numerous ailments [1,2,3]. The World Health Organization ranks physical inactivity as the fourth most common cause of death in the world, causing 6% of deaths [4]. In modern society, technical developments transform non-exercise physical activities into sedentary, screen-based activities and automation [5]. Sedentary activities, such as recreational sitting, reflected by television/screen time, are related to raised mortality and cardiovascular disease risk. Trying to compensate the sedentary lifestyle with occasionally or regularly vigorous exercise is difficult, partly due to inflammatory and metabolic risk factors [6]. Sedentary behaviour is increasingly described as a separate risk factor and not merely as a lack of physical activity or low levels of physical activity [7]. Prolonged sitting is a risk factor for all-cause mortality, independent of physical activity [8].

Sedentary lifestyles are also common among students. Students have been reported to be sitting on average around 10 h per day, of which more than 6 h are when they are at their university [9]. They are also sedentary during local transport and leisure time. Research from North America report that Canadian youths spend about 8.6 h of their waking hours in sedentary postures and in the United States the figure is 6–8 h [10,11]. Centers for Disease Control and Prevention [12] report that among people aged 18–24, only 41% of men and one-third of women obtain recommended levels of leisure time physical activity. From a public health aspect, there are therefore good reasons to find alternatives to sedentary teaching and learning situations. Several attempts in various settings have focused on addressing low levels of physical activity and sedentary situations by changing the indoor standard desk situation, adding equipment or modifying work postures [13,14,15], among others, but none of these addresses the situation for students or teachers in the act of teaching.

In lecture situations, there is an imbalance between teachers’ and students’ physical activity. Teachers are usually physically active during their lectures: they stand up, walk around while talking, point to different parts of presentations, write text on a board, etc. Students are at the same time normally physically passive, confined to their chairs during the same lecture. University settings provide students with a mainly sedentary design. Lectures are normally performed indoors with the professor standing and the students seated. This sedentary design has been taken for granted as the way learning and teaching occur: ‘*the desk as a technology for learning is a contrivance aimed at controlling movement and attention in whichever setting it inhabits. As such, it points to the premise underlying education in many cultures: to learn we must be still’* [16].

However, the design of the campus of the future focusses on shared space which is more intensively used and of better quality than at present [17]. The campus of the future supports ICT (Information and Communication Technologies)-based new ways of working that are adopted in modern office work including universities. These features contribute to reduce the campus carbon footprint and set the example for a new generation [17]. Thus, in line with social sustainability efforts, supporting increased physical activity among university students and staff is a public health goal for university campuses [18].

Blended learning, or hybrid courses, can improve the learning situation [19] but is no remedy for sedentary students if it only replaces lecture time with computer time. Due to developments in ICT, students and teachers can work wherever is best for them. New ways of working in terms of increased use of ICT are influencing how teaching and learning activities are organised [20], and also the design of the academic workplace. The focus is on less individual territory and more shared space [17]. The introduction of activity-based working in academic settings has raised the question of how to incorporate outdoors environments as a health-supporting work environment that could increase physical activity among university students and staff [21]. Previous research suggests that physical activity is a strategy to increase activity and reduce sedentary time in university-based settings [22].

As research results of the negative consequences of sedentary behaviour are becoming increasingly reliable, and the amount of sedentary behaviour on a population level is increasing, there is a need to explore alternative designs of the settings for teaching and learning [23]. Different types of workplace walking strategies influence university employee step counts and sitting times. Trying to motivate students to increase their level of physical activity, feedback and habits seem to be central components [24]. It is especially important to target adolescents during the transition to adulthood, when habits of physical activity are established, as during that time the decline of physical activity appears to accelerate. Intervention studies aimed at increasing levels of physical activity may have problems with providing long-term effects, and there are few studies that address or include sedentary time of students [25]. However, there are attempts to increase levels of physical activity through addressing both teachers and students. An intervention made opportunities for light intensive physical activity available in the classroom and arranged discussion sessions to provide guidance for the students to choose to augment their levels of physical activity [26].

There are reasons to be physically active also from a learning perspective. For example, ‘mind wandering’ or lack of attention is widespread in learning situations, where studies show that students can focus on average three to five minutes before they lose focus [27,28,29]. Increased physical activity can counteract this, as shown in a study where walking on a treadmill increased both attention and memory [30]. Furthermore, studies have shown that walking leads to increased creativity [31]. Spending time in nature is suggested to promote health and wellbeing, and to reduce work stress [32]. Previous research on university settings shows that workplace physical activity strategies to improve mental wellbeing and productivity among all employees should focus jointly on increasing physical activity and reducing sitting time [33,34]. Physical activity has been shown to have many beneficial effects on the brain, enhancing the health and survival of neurons, neurogenesis, the number of synapses, and plasticity. Among these effects are increased brain blood flow, brain grey matter volume (suggesting more neurons and synapses), volume in the frontal lobes (important for executive functions such as planning and concentration), volume in the hippocampus (important for learning and memory and increased brain connectivity, suggesting improved plasticity), brain volume and connectivity, and reduced age-related brain shrinkage [35]. There are good reasons to believe that increased physical activity is beneficial for both the brain in general and for learning.

However, one possible occasion where students could be physically active that is common in many educational settings is the seminar, as the purpose of many seminars is active discussion, and these seminars require neither technical aids for presentation nor intense note taking. Discussion seminars are difficult to lead productively [36] and previous studies have proposed allowing the students to run their own seminars in order increase attendance (which is correlated with academic performance [37], learning [38] and deepened understanding [39]) and to allow students to initiate conversations [40]. Using too large seminar groups may negatively influence the academically weak undergraduates [41] and increases the difficulties to give attention to the non-speaking students [40]. There are successful attempts to improve the seminar form with virtual seminars, although they cannot completely substitute for other forms of teaching in small groups [42] as they may weaken the social relations between students and lose the virtues of direct interaction. A balanced environment could provide a blend of both asynchronous and synchronous opportunities, which promote communication and collaboration among classmates and instructors.

As an alternative, we have in this study investigated the feasibility of seminars in walking form outdoors. We specifically examined how students experienced these seminars compared to traditional classroom-based seminars with the same purpose, their experiences of communication during the seminars, the quality, and how they felt after the seminars.

## 2. Materials and Methods 

In the present study, we combined these successful elements: allowing the students to run a part of the seminars themselves, using virtual seminars as a preparation for the physical seminars, and dividing the students into small groups. With these elements in place, it became possible to run the physical seminars outdoors, while walking.

The study object in this paper is the mandatory Programme Integrating course for the two 5-year engineering programmes in Media Technology and Computer Science at KTH (Kungliga Tekniska Högskolan—Royal Institute of Technology). The course facilitates students’ perception of connections between courses in their programme and provides a foundation for routines for lifelong learning, through reflection about the conditions of learning as well as by thematic exercises in contemporary cross-boundary subject areas from both within and outside the academy. The readings required before each seminar are not complicated academic texts, but are rather meant to give a brief introduction to the topic (often in the form of one or two videos or a podcast), and provides a backdrop for self-reflection, which is the main purpose of the seminars. The course is spread out over three years of the program and is studied in parallel with other courses [43].

A mandatory mentor-led seminar is given four times per academic year. Before the seminar, the students write a reflection document. Each document consists both of a theme section on a topic relevant to the education, which requires some type of reading and a reflection on this and the courses the students have followed since the last seminar. Examples of themes are ethics, study motivation and procrastination.

Students prepare for the seminar by reading and commenting on each other’s reflection documents using a social annotation system [44], similar to the virtual seminars reported by [42]. This results in well-prepared students at the seminar, where the theme and courses are discussed further. The requirement of the seminars is active participation in the discussions, and the debate climate is open and tolerant; it does not have the character of a quiz. However, the reflection documents are graded by the mentor on the four first levels of the reflection types identified by Hatton and Smith [45].

In spring 2014, as an experiment, one of the mentors led one of his groups in an outdoor walking seminar. The oral evaluation after the seminar was positive, weather permitting. For the next round of seminars, all four groups by the same mentor had walking seminars, this time evaluated by an anonymous online questionnaire (response frequency 85%) on their experiences, comparing the walking seminar to the previous indoor seminars with the same group in the same course. The results were in favour of the walking seminars: the students reported improved communication, sense of wellbeing, and quality of the seminar, and 83% reported they would like more walking seminars [46].

The seminar group gathers in the scheduled seminar room. A blind vote on whether the seminar should be held indoors or outdoors is performed. We have used both veto (if one student would like to stay indoors, all students stay indoors) and grouping based on the vote (if three to four students would like to stay indoors, they form their own subgroup while the rest is walking, divided into subgroups of two to four students). This handles challenging weather/poor clothing and physical ability issues. At the time of the study, the students had during that academic year on average participated in two indoor seminars and two outdoor seminars. While walking, the teacher walked between the groups to listen in and make notes of particularly interesting discussions or arguments. About two to three times during the seminar, the teacher gathered the subgroups to briefly discuss these particularly interesting issues with the whole group.

The questions in the survey in the pilot study were based on the feedback from the first group of students and a discussion with the course responsible teacher. For the present study, some of the questions were clarified (based on the comments and answers to the open-ended questions from students in the pilot study).

During the following academic year, the idea of walking seminars was disseminated to more teachers, and in the spring of 2015 a refined version of the survey used in 2014 was distributed to 176 students enrolled in either the Computer Science or the Media Technology programme who were likely to have participated in at least one walking seminar. Besides the above-mentioned programme integrating courses, 10 students were invited from the Degree Project course for Computer Science students. The group sizes varied from 3–5 for the Degree course, 6–9 for the Media course and 11–15 for the Computer Science course. All seminar questions had a seven-item Likert scale. 

As the participants’ views on physical activity could vary depending on how physically active they were otherwise; for this study, we also included the Active-Q questionnaire [47] to investigate the students’ physical activity levels. The survey was preceded by an invitation email and reminders were sent three times with eight days spacing. Students who participated in the Active-Q questionnaire were given feedback in the form of an email with both their individual activity level and the averages for the study group after the data collection was terminated. All participation in the study was voluntary and anonymous.

The nine interviewed teachers were teachers on at least one of the three courses mentioned above, or a Master’s level course in Philosophy of Science and Research Methodology for Computer Scientists. The interviews were structured and followed the same order as the questionnaire to the students. All interviews were recorded and transcribed. 

All subjects gave their informed consent for inclusion before they participated in the study. The study was conducted in accordance with the Declaration of Helsinki. As this work was made as a part of the course development and no sensitive data was collected, it did not require further ethics approval. 

## 3. Results

### 3.1. Questionnaire to Students

Of the 176 invited students, 131 answered (74.4%) the questionnaire on the walking seminars: 49 women and 82 men. The invited students were highly likely to have participated in a walking seminar. We know which groups of students participated, but we have not kept a record of students who temporarily switched to another group. A few of the invited students might not have participated in a walking seminar, and hence declined to answer.

**Figure 1:**
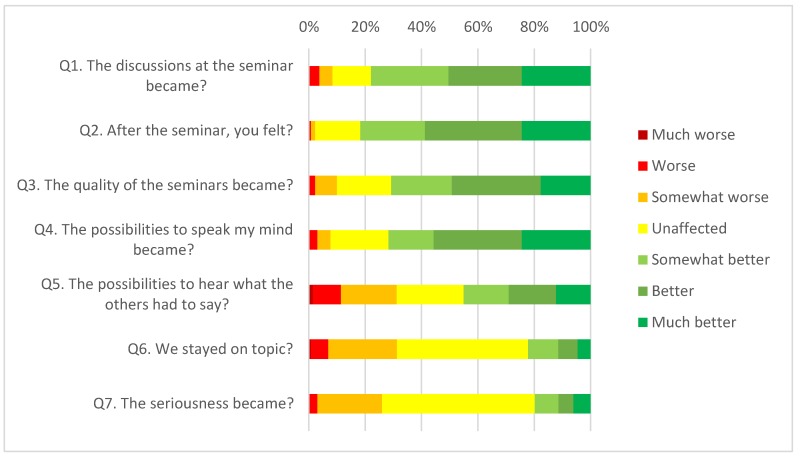
Perceived changes when comparing the previous indoor seminars to the outdoor seminars. *n* = 131.

In Table 1 and Figure 1, we can see the answers to the question “If you compare these walking seminars to the previous indoor, do you think that <Q*_n_*>”, where *n* is 1–7. In summary, 78% answered that the discussions improved, 82% felt better after the seminar, 71% thought that the quality of the seminars had improved, 72% thought the possibilities to speak had improved. Very few students reported deterioration on any of these questions. The possibilities to hear what others had to say was on average unaffected, but with individual variations, as were the score for stay on topic and seriousness. 

Comparing the Computer Science (CS) and the Media students, the CS students thought that their possibility to speak (Q4) improved significantly more (5.8 vs. 5.3, *t*-test *p*-value < 0.05) than the Media students (who also reported improvements). This effect might be explained by the larger group size for the CS students, making the effect of splitting up into smaller groups greater than for the media students.

Some answers in the general comment field were as follows:

I was way more active during the walk than I use to be when I mostly sit quiet and say some occasional thing at each seminar. Now I spoke out on each topic.

Incredible fun idea!

It becomes more relaxed which would benefit many seminars that otherwise are pretty stiff.

Definitely! I really cannot understand why <the university> not already has introduced this (walking seminars). Would become angry if anyone tried to counteract more walking seminars.

In Table 2 we can see that 89% appreciated being outdoors and 85% enjoyed the exercise. More than half of the students were very positive. Very few were negative. Some comments in the general comment field were as follows:

*Thumbs up for walking seminars! So nice to be active instead of sitting indoors!*

Walking seminars I think make more people discuss actively and it also become less stiff than in a seminar room. Besides it is healthy and fun to be outdoors and be active.

This mentioning of stiffness was confirmed in a separate question on whether the respondents found the walking seminar more relaxed; 91% said so, and 83% thought this was an improvement (16% were neutral). There is a Pearson correlation of 0.7 between those two questions, significant at the 0.01 level.

When asked about the ideal size of the subgroups, 3% answered two people, 59% three people, and 39% answered four people. Some of the students (*n* = 115) had experienced a reshuffle of the subgroups. Of these, 4% thought this was a bad idea, 59% were positive and the rest neutral. When asked whether their university should offer walking seminars in more courses, 9% were negative, 71% positive and the rest neutral.

### 3.2. Active-Q

Of the total 131 students who answered the seminar questionnaire, 115 (88%) also answered Active-Q, a detailed questionnaire on physical activity in general [47]. The total energy expenditure was measured as MJ/day; the average for the respondents was 11.5 MJ/day and the standard deviation was 2. The energy level is in line with a previous study of young adults [47,48]. Measured as hours at the four different levels of intensity, the average was sedentary: 10.8 h (SD 2.4), light: 4.8 h (SD 2.7), moderate: 1.2 h (SD 1.9) and vigorous 0.4 h (SD 0.6). 

There were no significant differences between registered genders on any of the seminar questions, but as expected there were differences in weight, height and 24-h energy expenditure.

Those who answered Active-Q felt significantly better after the seminar (Q2, 5.7 vs. 5.1, *t*-test *p*-value < 0.05) and also thought that the quality had improved more (Q3, 5.3 vs. 4.6, *t*-test *p*-value < 0.05) compared to those who did not answer Active-Q. However, there is no other correlation between any of the seminar questions and the level of physical activity, or Body-Mass Index (BMI). That is, the students were approximately equally positive to the walking seminars regardless of their normal physical activity level and BMI.

### 3.3. Teacher Interviews

The eight interviewed teachers were asked the same questions as the students, but answered orally. In Table 3, the interviewed teachers’ opinions on walking seminars are summarised. No one answered “Much worse”. We can see that the teachers’ opinions roughly matched those of the students. The teachers were positive for the three first questions (Q1–Q3) (average 5.4–5.9 on the 7-degree scale). Some citations to illustrate this follow. Regarding discussions (average 5.4):

In the ordinary context <indoor seminar>, the idea is that all should participate, but it becomes very much a communication between me and the student presently talking.

It results in more talk per student, and that should lead to better discussions.

Regarding how the teachers felt (average 5.9):

A day like this when you have had several seminars in a row in a small room, you become tired and get a headache. <after a walking seminar> Fresh and happy!

I sit, like everybody else, still and stare at a computer screen with vulture neck all day long, so it was great to get a break from that.

Regarding the quality of the seminar (average 5.5):

It worked well, and better, in this context <outdoors> probably because it defuses the stress of “now I am going to talk in front of the group”. When you walk and talk <...> it is less official.

After the first <outdoor> seminar there was a male student that said this was really great, because he was very shy and on top of it all you must speak English and he thought he would not have said anything if it had been an indoor seminar in a classroom.

When it comes to the possibilities to speak their mind, the average score was 5. The two teachers who answered “somewhat worse” elaborated on that:

The possibilities for me to speak my mind became somewhat worse, but that is really an improvement, because I have a tendency to talk too much during the seminars.

It became easier for me to shut up!

The possibilities to hear what others had to say was, on average, unaffected (4.1). However, just as with the possibilities to speak their mind, those who scored low did not consider this a disadvantage:

As seminar leader, I cannot hear everything because I walk between the groups so I no longer know everything nor hear everything, but that is not important as it is more important that the students talk.

The teachers thought the outdoor seminars were on a par with the indoor seminars when it came to staying on topic (3.9) and the seriousness (4.3). On the last account, one teacher explained:

Better. For some students, the difference was huge.

Two other observations from the interviews were that one teacher was really surprised by clear differences between the outdoor and indoor seminars, especially the positive qualitative differences. Another teacher proposed:

*Start with walking seminars in the beginning of program to enable all to get to know each other*. 

## 4. Discussion

We have performed an intervention study where students who were accustomed to traditional indoor seminars in a course were taken for a walk outdoors while performing a seminar. Surveying the students and interviewing the teachers, it was indicated that walking outdoors was in general perceived as an improvement on several accounts (discussions, quality, sense of wellbeing, ability to speak, ability to hear) and a slight deterioration on other accounts (staying on topic and seriousness). 

There are several limitations to this study. We have no objective measure of the results of these seminars, regardless of performing them indoors or outdoors. Nor do we know if it would be possible to perform similar outdoor seminars at other venues, as not all seminars are located next to a park as in the studied case. When it comes to the lack of objective measures, we make the assumption (as we did for the indoor seminars) that reflecting in your own words, discussing and arguing over a topic will lead to a deeper understanding. Regarding the location, it is obvious that noise pollution would be a serious impediment to hearing. The location we used was particularly suited for this purpose. However, it had its challenges: construction work at the university, park maintenance and outings by nearby schools and daycare facilities, but despite this, the students were positive. 

A 60-min indoor seminar with nine students will result, on average, in 7 min talking and 53 min listening for the students. A 60-min outdoor seminar with three sub-groups will result in 20 min talking and 40 min listening. This would be a tripling of the time talking but, in reality, when the discussions become intense in the sub-groups, much more will be said in the same time, which could explain the perception students report about being more active, more relaxed, and more open. The teachers’ role in these seminars is not teaching, but facilitating the discussion among the students; there is therefore no loss of “teaching time” for the teachers.

We have introduced two new items at the same time: walking and being outdoors. We have no way of determining whether the positive effects came from either aspect of the combination. One could envision a study where two groups of students were outdoors, one walking and one sitting, to separate these effects. However, due to the health benefits associated with walking, that outdoor walking “produced the most novel and highest quality analogies” [31] and spending time in nature is suggested to promote health and well-being, and to reduce stress [32], we would argue that if one has the option of conducting a seminar walking or sitting, one should always choose a walking seminar.

Students are the office workers of the future and getting them to adapt to healthy ways of working during their education may have important public health advantages. We acknowledge that a huge body of work and knowledge are developed, e.g., within the domain of occupational health when it comes to how to address low levels of physical activity and sedentary work-postures in many settings, including contexts in schools and universities. Nevertheless, we are motivated to continue to explore the benefit of introducing walking outdoors as an alternative to redesigning classrooms. Previous research suggests that physical activity should be recognised as a legitimate activity at work, embedded within the university culture and endorsed using a top-down approach. Further, it is important to recognise physical activity as a legitimate health-promoting activity that is supported and encouraged during working hours [22]. 

Based on this study and our experience of walking seminars, we argue that similar walking seminars could be used in other venues, as long as the goal of the seminar is the discussion and the deepened understanding of the discussed topics that follows (in contrast to information distribution). We have performed walking seminars with classes of up to 40 students at the same time. The ideal subgroup size seems to be three. Four is possible, but requires wide walking paths and strong voices. 

These seminars where themes are only discussed is one subgroup of all seminars. We therefore aim to include more types of seminars in the future, for example, seminars that do require notes to be taken and studies on requirements for those tools that must be added to those seminars. Also, the method described here would benefit from being tested in different venues, e.g., ordinary city parks.

## 5. Conclusions

We have conducted a study of the possibility of conducting seminars while walking outdoors. This seminar form was made possible by using three previously identified elements for improving seminars: virtual seminars, small groups, and student involvement. The study shows that a sense of wellbeing can be achieved at the same time as the perceived quality of the seminars increased according to both the students and the teachers who led the seminars. Including outdoor walking in similar ways into ordinary workdays could have many possible benefits both for health and learning [27,28,29,30,31,32,33,34,35]. Encouraging people to be more physically active is not enough [11,12]. In this study, we have designed a learning activity that includes light to moderate physical activity. The purpose has been to remedy the sedentary manner of learning and explore how walking would be perceived by students and their teachers.

## Figures and Tables

**Table 1:** Perceived changes when comparing the previous indoor seminars to the outdoor seminars. *n* = 131.

	Much Worse	Worse	Somewhat Worse	Unaffected	Somewhat Better	Better	Much Better
Q1: The discussions at the seminar became?	0%	3.8%	4.6%	13.7%	27.5%	26%	24.4%
Q2: After the seminar, you felt?	0%	0.8%	1.5%	16%	22.9%	34.4%	24.4%
Q3: The quality of the seminars became?	0%	2.3%	7.7%	19.2%	21.5%	31.5%	17.7%
Q4: The possibilities to speak my mind became?	0%	3.1%	4.6%	20.6%	16%	31.3%	24.4%
Q5: The possibilities to hear what the others had to say?	1.5%	9.9%	19.8%	23.7%	16%	16.8%	12.2%
Q6: We stayed on topic?	0.8%	6.1%	24.4%	46.6%	10.7%	6.9%	4.6%
Q7: The seriousness became?	0%	3.1%	22.9%	54.2%	8.4%	5.3%	6.1%

**Table 2:** Opinions on being outdoors and exercise. *n* = 130.

What Did You Think of	Very Bad	Bad	Not so Good	Nothing in Particular	Pretty Good	Good	Very Good
being outdoors?	0	3.1	2.3	5.4	9.2	21.5	58.5
the exercise?	0	0	3.1	11.5	12.3	22.3	50.8

**Table 3:** Teachers’ perceptions of changes compared to the previous indoor seminar.

	Worse	Somewhat Worse	Unaffected	Somewhat Better	Better	Much Better
Q1: The discussions at the seminar became?			2	1	5	
Q2: After the seminar, you felt?				4	1	3
Q3: The quality of the seminars became?		1	1		5	1
Q4: The possibilities to speak my mind became?		2		2	2	1
Q5: The possibilities to hear what the others had to say?	1	2	2		1	1
Q6: We stayed on topic?		2	5	1		
Q7: The seriousness became?		1	5	1	1	
